# A blind randomized validated convolutional neural network for auto‐segmentation of clinical target volume in rectal cancer patients receiving neoadjuvant radiotherapy

**DOI:** 10.1002/cam4.4441

**Published:** 2021-11-23

**Authors:** Yijun Wu, Kai Kang, Chang Han, Shaobin Wang, Qi Chen, Yu Chen, Fuquan Zhang, Zhikai Liu

**Affiliations:** ^1^ Department of Radiation Oncology Peking Union Medical College Hospital Chinese Academy of Medical Sciences & Peking Union Medical College Beijing China; ^2^ MedMind Technology Co., Ltd. Beijing China

**Keywords:** deep learning, rectal cancer, neoadjuvant radiotherapy, convolutional neural network, clinical evaluation

## Abstract

**Background:**

Delineation of clinical target volume (CTV) for radiotherapy is a time‐consuming and labor‐intensive work. This study aims to propose a novel convolutional neural network (CNN)‐based model for fast auto‐segmentation of CTV. To evaluate its performance and clinical utility, a blind randomized validation method was used.

**Methods:**

Our proposed model was based on the generally accepted U‐Net architecture using computed tomography slices with CTV contours delineated by experienced radiation clinicians from 135 rectal patients receiving neoadjuvant radiotherapy. The Dice similarity coefficient (DSC) and 95th percentile Hausdorff distance (95HD) were used to measure segmentation performance. The validated dataset of additional 20 patients for clinical evaluation by 10 experienced oncology clinicians from 7 centers was randomly and blindly divided into two groups for clinicians' scoring and Turing test, respectively. Second evaluation was performed with different randomization after 2 weeks.

**Results:**

The mean DSC and 95HD values of the proposed model were 0.90 ± 0.02 and 8.11 ± 1.93 mm for CTV of rectal cancer patients, respectively. The average time for automatic segmentation in the validation groups was 15 s per patient. By clinicians' scoring, the AI model performed better than manually delineating, though the differences were not significant (Week 0: 2.59 vs. 2.52, *p* = 0.086; Week 2: 2.55 vs. 2.47, *p* = 0.115). Additionally, the mean positive rates in the Turing test were 40.5% in Week 0 and 45.2% in Week 2, which demonstrated the great intelligence of our model.

**Conclusions:**

Our proposed model can be used clinically for assisting contouring of CTVs in rectal cancer patients receiving neoadjuvant radiotherapy, which improves the efficiency and consistency of radiation clinicians' work.

## Graphical abstract

This study first developed an auto‐segmentation system using U‐Net deep learning architecture in patients receiving neoadjuvant radiotherapy and obtained great measurement performance. Besides, we first reported a blind system for clinical evaluation, including clinicians' scoring and Turing test by multicenter radiologists, which can eliminate inter‐ and intra‐observer discrepancy as much as possible. Our proposed methods may be helpful for the assessment of AI‐assisted models in clinical practice.

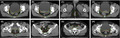

## 1 BACKGROUND

Rectal cancer remains to be one of the most common and deadliest malignancies worldwide.^1^ Neoadjuvant radiotherapy has been proved to play a critical role in the treatment of locally advanced rectal cancer, which demonstrated better local control rates than surgery alone.^2^ In the process of radiation therapy, the delineation of clinical target volume (CTV) and organs at risk (OARs) is one of the most essential steps. In spite of several guidelines for the contouring delineation on rectal cancer patients,^3^, ^4^, ^5^ it still remains difficult for all delineated slices to be precise and acceptable. Inappropriate contouring for CTV and OARs would reduce therapeutic advantages and increase the risk of radiation exposure of normal issues, respectively. Additionally, there is still lack of delineation consensus considering the inevitable and significant intra‐ and inter‐observer inconsistence between radiation oncologists and centers.^6^ Thus, innovations on contouring are required to improve its accuracy and reproducibility, and to decrease intra‐ and inter‐observer discrepancy.

Manually delineating regions of interest (ROIs) slice by slice on computed tomography (CT) images is a time‐consuming and labor‐intensive work for radiation oncologists. More applications related to radiation therapy have been conformed for diseases in recent years, and thus radiation clinicians are required to accurately complete the delineation of ROIs in a short time. To improve contouring efficiency, automatic segmentation assisted by state‐of‐the‐art tools was outlined with the advancement of multidisciplinary concepts and techniques. Artificial intelligence (AI), especially deep learning algorithms, has demonstrated extraordinary feasibility in medicine and may be able to bring revolutionary changes in the workflow of radiation therapy.^7^, ^8^, ^9^

It was reported that a series of studies had developed automatic contouring models using convolutional neural network (CNN), which predominated in the computer vision field for image segmentation.^10^, ^11^, ^12^, ^13^ Kuo et al. developed a deep dilated CNN (DDCNN)‐based model for segmentations of the rectal cancer patients' CTV with a mean DSC value of 0.877, showing 3.8% higher than that of U‐Net they used.^14^ Both of the two methods are based on two‐dimension convolutions, whereas Rasmus et al. designed a three‐dimension V‐net architecture that derived from the U‐Net, with a higher DSC reaching 0.90 more than U‐Net (0.84) and DDCNN (0.87).^15^ Subsequently, Ying et al. developed a DeepLabv3+ architecture for delineating CTV in rectal cancer patients that received postoperative radiotherapy, which demonstrated similar DSC values as previous studies, thought it performed significantly better than the U‐Net‐derived ResUNet in quantitative parameters.^16^ Though these previous studies reported high quantitative performance CNN‐based contouring models for rectal cancer, clinical evaluation was not further validated in these studies, and almost none of them had been tested in the real clinical circumstances. Furthermore, there are still no commonly accepted methods and criteria for clinical practice.

Compared to other organs, the delineation of rectum CTV should be more challenging for the complexity of pelvic compartments. In most cases, there is actually lack of clear boundaries of rectum CTV, and thus the conventional methods of contouring that relied on the images’ gray‐level are limited. In contrast, the novel CNN can extract and identify significant texture features of high levels by learning from a large database of images with artificial marked contours, which would delineate more accurate and applicable ROIs. In the present study, we developed an auto‐segmentation model based on the classical U‐Net architecture for neoadjuvant radiotherapy of rectal cancer patients. To assess its clinical accuracy and utility, the blind randomized validated tests were also performed by 10 experienced oncology clinicians from seven centers.

## 2 METHODS

### 2.1 Data source

Computed tomography images from 135 consecutive patients (training set: 122 cases; validation set: 13 cases) with locally advanced rectal cancer that received neoadjuvant radiotherapy at Peking Union Medical College Hospital between July 2018 and August 2019 were included to develop the deep learning‐based auto‐segmentation model. All patients' ground truth (GT) CTVs were manually delineated by radiation oncologists with more than 10‐year experience. This study has been approved by the Ethics Committee of Peking Union Medical College Hospital and all patients have signed informed consent.

### 2.2 Simulation

Patients were in the supine position immobilized with thermoplastic trunk mask. They received a contrast‐enhanced CT scan with a Big‐Bore CT (Philips). Images were acquired from upper bound of L1–2 cm below the lower edge of ischial tuberosity.

### 2.3 CTV definition

According to the RTOG consensus, the CTV includes internal iliac, presacral, and perirectal nodal regions.

### 2.4 Network model

**FIGURE 1:**
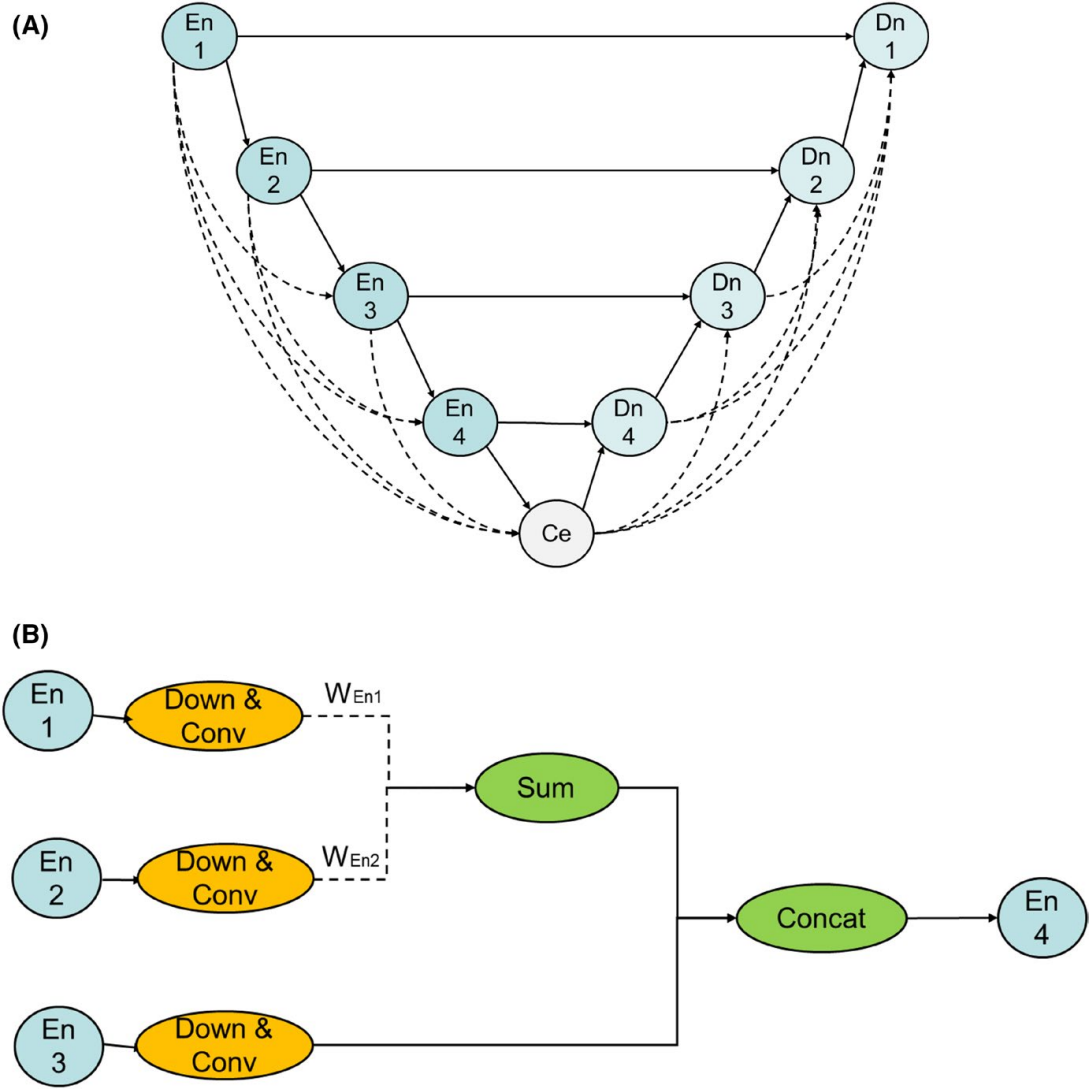
Development of our U‐Net architecture. En is encoder block. Down and Conv are down sample and convolution layers. *W*EN is connection weight responding to a specific connection

U‐Net is a successful architecture in medical image segmentation due to its skip connections which combines the high‐level semantic feature maps from the decoder and low‐level detailed feature maps from the encoder. In our new model (Figure 1A), we take the advantage of U‐Net, and augment the combination of multiscale feature by adding some skip connections with learnable weights. In encoder path, the added connections connect each layer to every other layer in a feed‐forward fashion. In decoder path, similar connections added as well. Furthermore, we propose a scheme to learn to connect/disconnect the added connections on its importance (Figure 1B). After weight of each connection is trained, we only keep the corresponding connections with weights larger than a predefined threshold. With GTX 1080 GPU, the final model was constructed via more than 50 circles for identifying the optimal one that demonstrated the lowest validation loss score.

Following the development of our proposed model, the Dice similarity coefficient (DSC) and 95th percentile Hausdorff distance (95HD) were used to measure its segmentation performance. The DSC is defined as shown in Equation (I), while 95HD is defined as shown in Equation (II–IV).

$$\text{DSC}\left(A,\;B\right)=\frac{2\left|A\cap B\right|}{\left|A\right|+\left|B\right|},\left(I\right)$$

where *A* represents the GT contours manually delineated by clinicians and *B* denotes the auto‐segmentations generated by AI. *A*∩*B* means the intersection of *A* and *B*. The values of DSC range from 0 to 1, where 0 represents no intersection between *A* and *B* and 1 means perfect overlapping.

95HD(A,B)=max(h(A,B),h(B,A),95th),(II)

$$h\left(A,\;B\right)=\max_{a\in A}\min_{b\in B}\left|\left|a‐b\right|\right|,\left(III\right)$$

$$h\left(B,\;A\right)=\max_{b\in B}\min_{a\in A}\left|\left|b‐a\right|\right|,\left(IV\right)$$

where ||.|| is the Euclidean norm of the points in *A* and *B*. HD can be described as the maximal value of the shortest distance between the point sets of *A* and *B*.

### 2.5 Clinical evaluation

**FIGURE 2:**
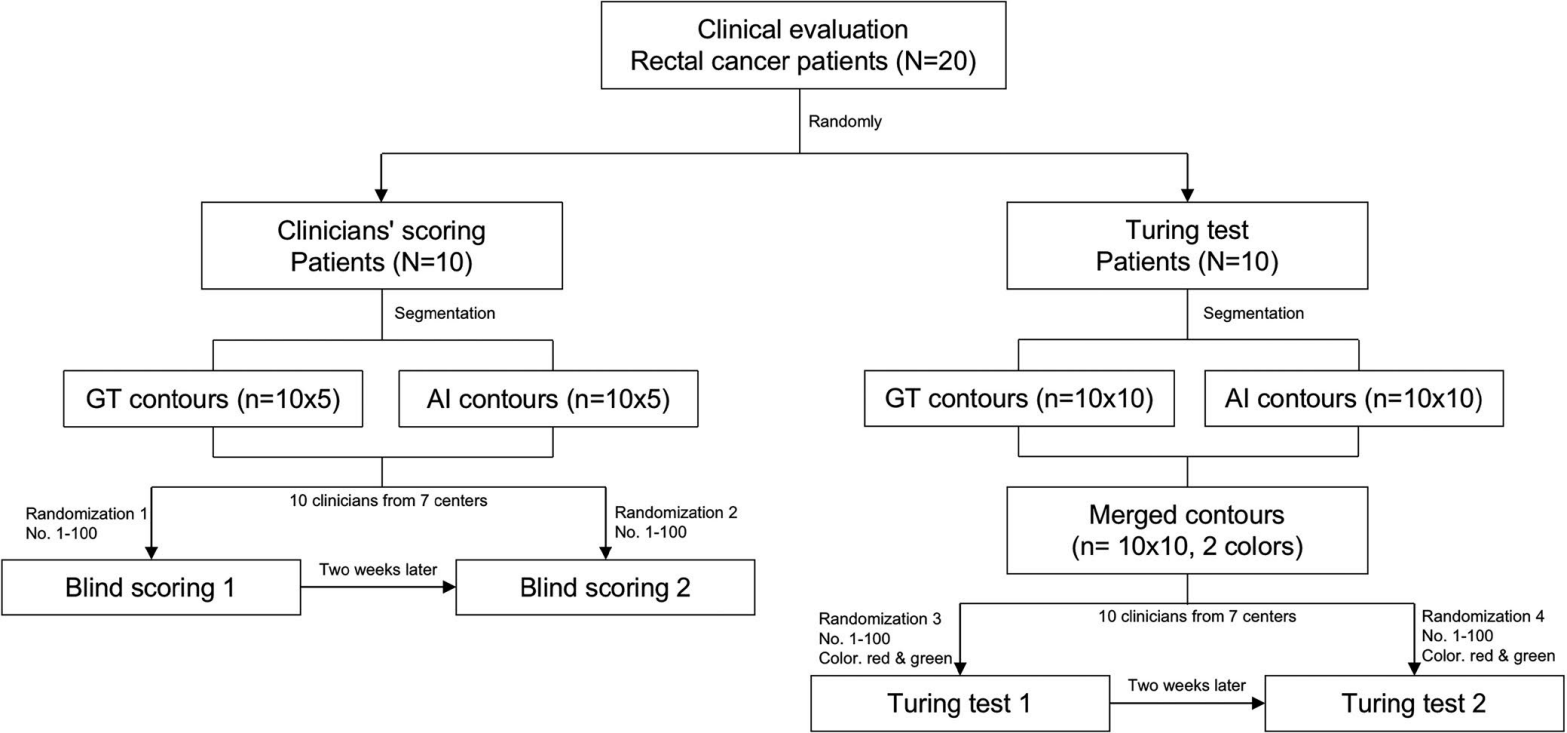
Flow chart for clinicians' scoring and Turing test. AI, artificial intelligence; GT, ground truth

To further assess clinical practicality of the proposed model, we prospectively enrolled another consecutive 20 patients who diagnosed with locally advanced rectal cancer for neoadjuvant radiotherapy between November 2019 and December 2019. Patients who had a history of pelvic surgery, other malignancy, or chronic diseases were excluded. These patients were randomly divided into two groups as a ratio of 1:1 for clinicians' scoring and Turing test, respectively, by the statistician (Figure 2).

**FIGURE 3:**
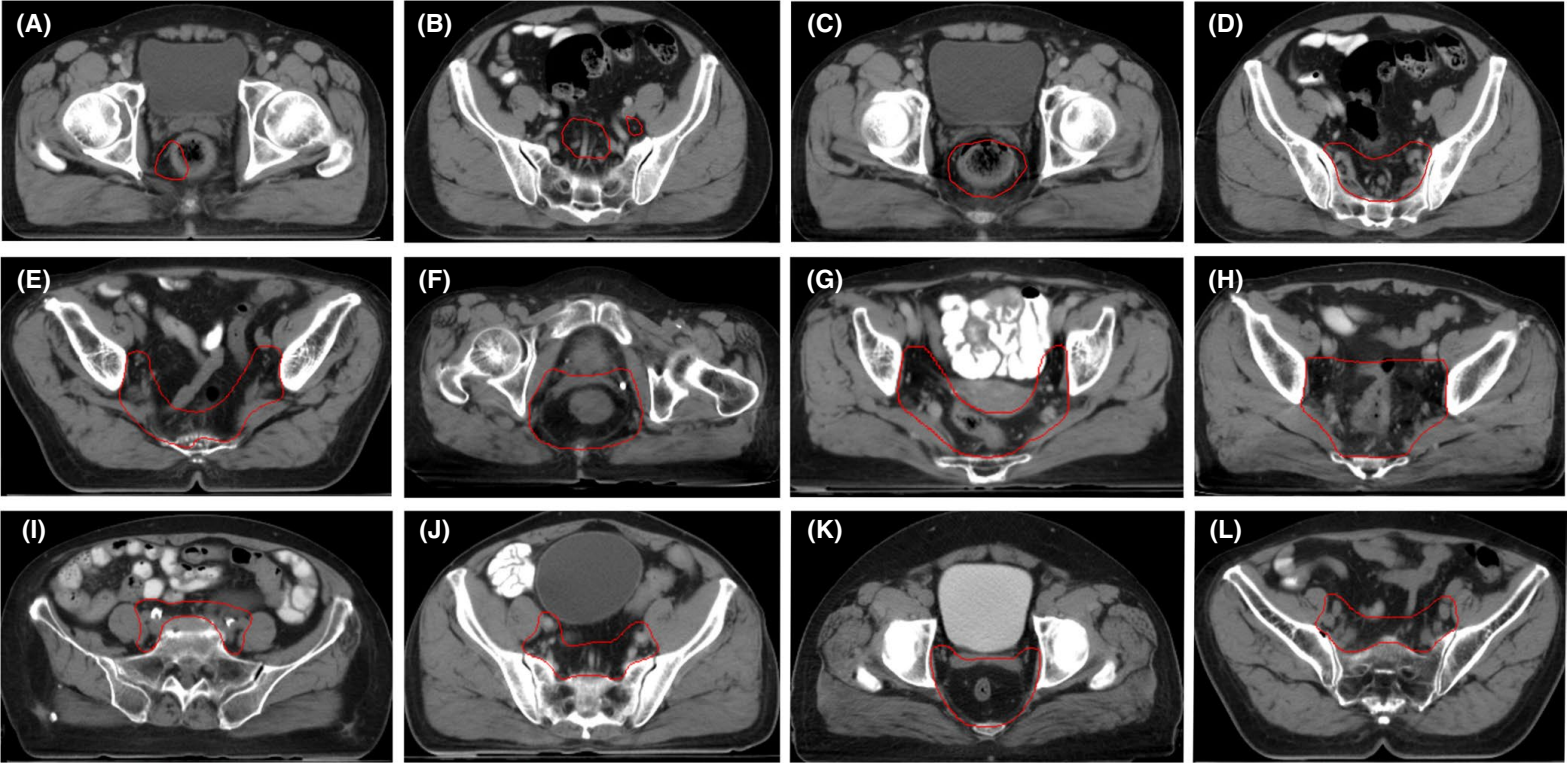
Sample contours for clinicians' scoring in patients with rectal cancer receiving neoadjuvant radiotherapy. Rejected (0 point): (A, B); Major revision (1 point): (C, D); Minor revision (2 points): (E–H); and Totally accepted (3 points): (I–L)

For clinicians' scoring, five GT and five AI contours of each patient were randomly extracted. Then a total of 100 contours (GT: 5 × 10 contours; AI: 5 × 10 contours) were randomized by the statistician (Figure 2), and were blindly and independently scored by 10 clinicians from seven centers. After 2 weeks, all contours were evaluated again by clinicians with differently randomized coding. The scoring criteria are as follows: 0 point (Rejected: The contour is unacceptable and requires redrawing), 1 point (Major revision: The contour requires significant revision, and treatment planning should not proceed without correction), 2 points (Minor revision: The contour should be revised with a few minor edit but has no significant effect on treatment without correction), and 3 points (Totally accepted: Perfect and completely acceptable for treatment). The scoring samples are shown in Figure 3.

**FIGURE 4:**
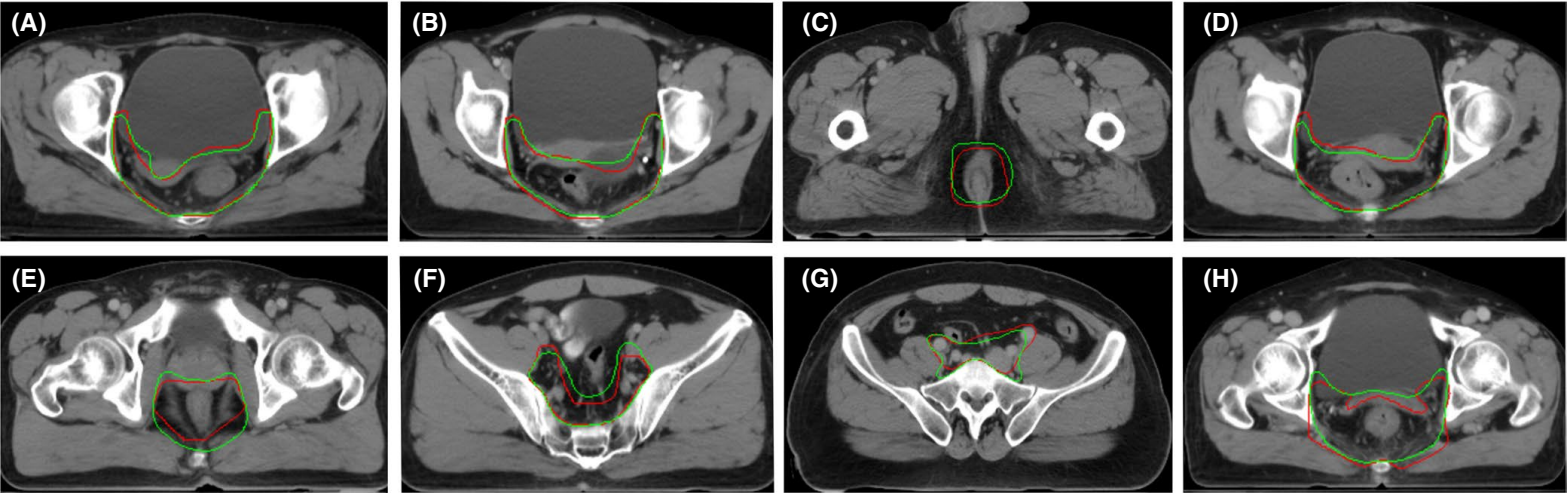
Sample slices for Turing test in patients with rectal cancer receiving neoadjuvant radiotherapy. Red: artificial intelligence (AI) contour; Green: ground truth (GT) contour. (A–D): AI performs better than GT; (E–H): AT performs worse than GT

Turing test is an important measure of how “intelligent” an AI model can be. In our test, clinicians were shown two contours overlaid in each CT slice (one was generated by AI and the other one was manually delineated, but which color represented AI or GT was unknown to clinicians). A total of 100 CT slices with merged GT and AI contours from 10 patients were tested (Figure 2). The two contours in each slice were randomly marked with two colors (red and green) by the statistician. Finally, all clinicians would independently give a comment which “color” was better for radiation therapy. Similarly, all slices were evaluated again by clinicians with the different randomization of both code and color 2 weeks later. If the AI model performs better than the manually delineated among more than 30% of slices, it can be considered to pass the Turing test and to be intelligent. Some typical samples are shown in Figure 4.

### 2.6 Statistical analysis

In this study, all randomizations and statistical analyses were performed by the statistician and were unknown to all clinicians. DSC and 95HD values were expressed as mean with standard deviation. The difference between the two randomized groups of patients for clinicians' evaluation and Turing test was compared by the Mann–Whitney *U* test. The agreement of clinicians' evaluation between the time interval of 2 weeks was assessed using the Kappa test (Kappa value ≥ 0.2 can be considered with an acceptable range of consistency), and the distribution consistency of Turing test was compared by the McNemar’s test. *p* < 0.05 was considered statistically significant.

## 3 RESULTS

### 3.1 Segmentation performance

The values of DSC and 95HD for each patient from our proposed model are shown in Table 1. The mean DSC values plus standard deviation of the two randomized validated groups for clinicians' evaluation and Turing test were 0.91 ± 0.02 and 0.89 ± 0.02 (*p* = 0.113), while the mean 95HD values were 8.59 ± 1.98 and 8.97±1.86 (*p* 0.284), respectively. The average time for automatic segmentation in the validation groups was 15 s per patient, compared with about 45–60 min for manual work in the clinical practice.

**TABLE 1:** DSC and 95HD values of our proposed model in the validation dataset of patients for clinicians' scoring and Turing test

Evaluation	Patient	DSC	95HD
Clinicians' evaluation	1	0.91	9.51
	2	0.89	8.13
	3	0.89	9.02
	4	0.93	5.94
	5	0.93	7.05
	6	0.93	5.97
	7	0.88	11.68
	8	0.89	10.46
	9	0.93	7.60
	10	0.90	10.50
Mean ± SD	−	0.91 ± 0.02	8.59 ± 1.98
Turing test	11	0.88	9.62
	12	0.84	10.08
	13	0.89	8.46
	14	0.88	9.79
	15	0.92	5.02
	16	0.91	5.47
	17	0.89	8.11
	18	0.90	6.98
	19	0.90	7.09
	20	0.91	5.76
Mean ± SD	−	0.89 ± 0.02	8.97 ± 1.86
*p* value	−	0.113	0.284

Abbreviations: 95HD, 95th percentile Hausdorff distance; DSC, Dice similarity coefficient.

### 3.2 Clinicians' scoring

**FIGURE 5:**
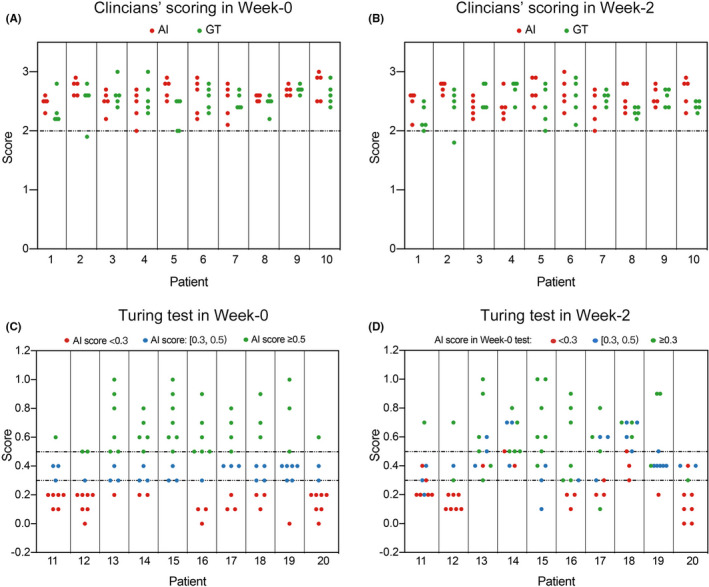
(A, B) Mean score of 10 clinicians for each contour. (C, D) Mean score of 10 clinicians for each slice in Turing test. AI, artificial intelligence; GT, ground truth

To verify our proposed model's clinical usefulness, 10 oncology clinicians from seven centers with more than 10 years of clinical experience blindly evaluated the segmented contours and scored them on 4 levels: 0 point (Rejected), 1 point (Major revision), 2 points (Minor revision), and 3 points (Totally accepted). The evaluation results are demonstrated in Table 2. Those with score ≥ 2 were defined to be suitable for clinical application. According to the evaluation by clinicians in Week 0, 94.6% of AI contours and 94.0% of GT contours were scored as ≥2 points, while 65.0% and 58.0% were 3 points, which could be directly used for radiation without any revision, respectively. More specifically, the AI group's mean scores were better than the GT’s, though there was no significant difference (Week 0: 2.59 vs. 2.52, *p* = 0.086; Week 2: 2.55 vs. 2.47, *p* = 0.115; Table 2). To further evaluate the clinical practice, we calculated the mean value of scores from all clinicians for each contour (Figure 5A,B). None of AI contours had mean score less than 2 points in both two scoring evaluations (Week 0 and Week 2). The Kappa value for each clinician was obtained (Table 2) and most of them demonstrated acceptable consistency among results between the 2‐week interval.

**TABLE 2:** Clinicians' scoring for AI and GT contours

Clinician	A		B		C		D		E		F		G		H		I		J		Mean (%)	
Score	AI	GT	AI	GT	AI	GT	AI	GT	AI	GT	AI	GT	AI	GT	AI	GT	AI	GT	AI	GT	AI	GT
Week 0																						
0	0	0	0	0	1	1	0	0	0	0	0	0	0	0	0	0	0	0	0	0	0.1 (0.2)	0.1 (0.2)
1	1	1	0	0	7	11	0	0	0	3	0	0	1	1	17	13	0	0	0	0	2.6 (5.2)	2.9 (5.8)
2	6	13	25	22	27	27	3	11	37	36	3	8	12	15	11	20	18	23	6	5	14.8 (29.6)	18.0 (36.0)
3	43	36	25	28	15	11	47	39	13	11	47	42	37	34	22	17	32	27	44	45	32.5 (65.0)	29.0 (58.0)
Mean score	2.84	2.70	2.50	2.56	2.12	1.96	2.94	2.78	2.26	2.16	2.94	2.84	2.72	2.66	2.10	2.08	2.64	2.54	2.88	2.90	2.59	2.52
*p* value	0.095		0.55		0.247		0.022		0.347		0.112		0.521		0.846		0.312		0.75		0.086	
Week 2																						
0	0	0	0	0	0	0	0	0	0	0	0	0	0	0	15	10	0	0	0	0	1.5 (3.0)	1.0 (2.0)
1	0	1	0	0	1	4	0	0	4	8	0	0	0	0	8	16	0	0	0	0	1.3 (2.6)	2.9 (5.8)
2	7	7	27	32	25	34	4	5	37	36	3	13	11	13	11	10	15	14	12	12	15.2 (30.4)	17.6(35.2)
3	43	42	23	18	24	12	46	45	9	6	47	37	39	37	16	14	35	36	38	38	32.0 (64.0)	28.5 (57.0)
Mean score	2.86	2.82	2.46	2.36	2.46	2.16	2.92	2.90	2.10	1.96	2.94	2.74	2.78	2.74	1.56	1.56	2.70	2.72	2.76	2.76	2.55	2.47
*p* value	0.751		0.312		0.008		0.728		0.181		0.007		0.641		0.989		0.826		1.000		0.115	
Kappa value	0.613		−0.029		0.240		0.463		0.158		0.361		0.115		0.155		0.264		0.495		–	–
*p* value^a^	<0.001		0.766		0.001		<0.001		0.04		<0.001		0.229		0.004		0.006		<0.001		–	–

Abbreviations: AI, artificial intelligence; GT, ground truth.

^a^ Kappa test.

### 3.3 Turing test

Another 10 patients that met our criteria were enrolled for Turing test and 10 slices were randomly extracted from each of them (Figure 1). For each slice, the AI and GT contours were independently delineated and were then merged with different and random colors, which were blind to all clinicians. The slice would be recorded as positive when its AI contour performed better than the GT contour. As shown in Table 3, the positive rates of all clinicians met the intelligence criterion of AI model (more than 30%). The mean positive rates for Week 0 and Week 1 were 40.5% and 45.2%, respectively, and there was also acceptable consistency between 2 weeks via the McNemar’s test.

**TABLE 3:** Turing test

Clinician	A	B	C	D	E	F	G	H	I	J	Mean positive rate
Week 0											
Positive	39	41	30	32	42	50	37	42	39	53	40.5%
Negative	61	59	70	68	58	50	63	58	61	47	
Week 2											
Positive	41	48	41	49	44	46	42	46	52	43	45.2%
Negative	59	52	59	51	56	54	58	54	48	57	
*p* value	0.059	0.334	0.008	0.096	0.198	0.76	0.05	0.281	0.452	0.752	–

Each slice was also scored as zero or one point (for the slice, if a clinician think AI contour is better than GT, it will get one point, otherwise zero point). The mean score for each slice has been calculated (Figure 5C,D). Most of slices had score ≥0.3, which means there were at least three clinicians thought the AI contour in this slice was better than GT. Nearly half of slices were scored ≥0.5, indicating clinicians cannot distinguish between AI and GT contours in these slices.

## 4 DISCUSSION

In the past decade, there has been encouraging advancements with regards to radiation therapy. CTV delineation is a key step in the planning of radiotherapy delivery that mainly relies on the time‐consuming manual work. Additionally, the inter‐ and intra‐observer variability cannot be ignored, which are related to tumor control and prognosis. However, the emerging techniques in recent years were devoted to the improvement of delineation efficiency and contouring standardization, and the CNN for automatic segmentation based on deep learning performed best. Radiation therapy has been considered to be the effective neoadjuvant treatment for rectal cancer preoperatively or postoperatively.^17^ In our study, we first developed a U‐Net‐based CNN model for automatically contouring CTVs in rectal cancer patients receiving neoadjuvant radiotherapy. Furthermore, the blind evaluation and Turing test by 10 experienced clinicians from different centers were also first designed to assess the model's clinical accuracy and usefulness. The mean DSC values of the two randomized validated groups were 0.91 ± 0.02 and 0.89 ± 0.02, which were similar to the previous studies on rectal cancer patients using different CNN architectures.^14^, ^15^, ^16^ However, all of these studies focused on mathematics quantitative compares, and none of them performed clinical evaluations.

Given the complexity of pelvic compartments and the ambiguous boundaries between rectums and others, delineating high‐quality CTVs is a kind of challenging work, which requires advanced AI techniques for assistance. The U‐Net architecture we used has demonstrated encouraging application foregrounds in auto‐contouring of medical images.^18^ Our proposed model was constructed using the dataset of image slices from 135 rectal cancer patients receiving neoadjuvant radiotherapy. Generally, the most common evaluation parameters for the CNN model were DSC and 95HD. Another 20 patients for clinical validation were randomly divided for clinicians' scoring and Turing test. The values of DSC and 95HD indicated great contouring performance of the model (Table 1). However, the two parameters could only describe mathematical performance rather than values of clinical application, and thus clinicians' evaluation is required. Here, we first designed a multicenter blind system with the involvement of clinicians' scoring and Turing test for further clinical assessment of our proposed model. Ten experienced radiation oncologists from seven centers participated in examining the clinical scoring. First, CT slices with AI or GT contours were anonymously scored (Table 2), including 0 point (Rejected), 1 point (Major revision), 2 points (Minor revision), and 3 points (Totally accepted). To avoid intra‐observer bias, another evaluation was also performed after 2 weeks with different randomized coding among these CT slices.

According to all clinicians, most of AI contours were acceptable (score ≥ 2; Table 2). Furthermore, the mean scores of AI group were higher than those of GT group, though there was no significance (2.59 vs. 2.52, *p* = 0.086), showing a great clinically delineating performance of our model. At the same time, our study also indicated the intra‐ and inter‐observer variability of CTV contouring. In Week 0, the whole team of clinicians evaluated almost all contours acceptable except clinician C (inacceptable: AI 16% vs. GT 24%) and H (inacceptable: AI 34% vs. GT 26%), but no significant difference between AI and GT groups was observed via their scoring. It could be inferred that the multi‐evaluator design of multicenter could eliminate inter‐observer variance as much as possible. In addition, the scoring of slices with contours is a subjective process and intra‐observer variance or time heterogeneity cannot be ignored (the same evaluator may give different scores for the same contour). Thus, another evaluation with different randomization for slices was performed by each oncologist after 2 weeks. A similar result was obtained (mean score: AI, 2.55 vs. GT, 2.47, *p* = 0.115). The Kappa test was used to compare the consistency of these two evaluations. In spite of low‐level consistency (Kappa value < 0.2) in accordance with some oncologists’ evaluation (clinician B, E, G, and H), AI group showed a greater mean score and even had significantly higher scores by clinician C’s and F’s than GT.

Besides, the mean score for each contour was obtained from clinicians' scoring (Figure 5). Most of slices had score ≥ 0.3, which means there were at least three clinicians thought the AI contour in this slice was better than GT. Nearly half of slices were scored ≥ 0.5, indicating clinicians cannot distinguish between AI and GT contours in these slices. Meanwhile, these results also showed some objective inter‐ and intra‐clinician differences for CTV contouring. Above all, after eliminating the effects of intra‐ and inter‐observer variance by the blind randomized design for evaluation, our proposed model can be applied well in the clinical practice for automatic contouring.

Additionally, we also performed Turing test for assessing the intelligence of our model. The slice would be recorded as positive when its AI contour performed better than the GT contour. When positive rate ≥30%, the model can be regarded as intelligent. In our study, the positive rates of all clinicians were larger than 30%. The mean positive rates were 40.5% in Week 0 and 45.2% in Week 2 (Table 3). The mean score for each slice has been calculated (Figure 5C,D). Most of slices had score ≥0.3, which means there were at least three clinicians thought the AI contour in this slice was better than GT. Nearly half of slices were scored ≥0.5, indicating clinicians cannot distinguish between AI and GT contours in these slices. Meanwhile, though the consistency test showed that most clinicians maintained insignificant discrepancy between 2 weeks, the results also showed some objective inter‐ and intra‐clinician differences for CTV contouring. Therefore, after trying to eliminate bias, our proposed model can meet the criteria of AI and would provide intelligent assistance for automatic segmentation.

Beside great contouring performance, our CNN‐based model takes superior advantages in time saving. Previously, manually delineating CTVs of one rectal cancer patients may require more than dozens of minutes. However, it only takes several seconds for the CNN model we developed to finish the work. With its assistance, the CTVs can be used clinically after examination and revision by radiation oncologists, which would decrease the consumed time to less than 10 min and thus greatly improve work efficiency.

Several limitations should be considered in our study. First, though clinical evaluation was conducted by multicenter clinicians, the data of CT slices and patients originated from the single center and the trained model might not be suitable for all centers. Thus, we aim to develop universally applicable transfer learning‐based models in the future studies, which can adjust segmentation performance based on individual clinician's or center's characteristics using a small set of trained samples.^19^ Second, the scoring evaluation by oncologists from seven centers is subjective and certain bias could not be totally avoided, and inter‐ and intra‐observer variance cannot be completely eliminated.

## 5 CONCLUSIONS

In conclusion, our study demonstrates that accurate auto‐segmentation of CTVs can be realized by the CNN model in rectal cancer patients receiving neoadjuvant radiotherapy. Clinicians' scoring and Turing test by 10 experienced radiation oncologists indicates that our model can be applied clinically to provide intelligent assistance for CTV contouring and improve efficiency. Our first proposed evaluation methods may provide references for AI models to assess clinical practice.

## CONFLICT OF INTEREST

Shaobin Wang, Qi Chen, and Yu Chen were employed by the company MedMind Technology Co., Ltd. The remaining authors declare that the research was conducted in the absence of any commercial or financial relationships that could be construed as a potential conflict of interest.

## AUTHORS' CONTRIBUTIONS

Zhikai Liu and Fuquan Zhang contributed to the conception of the study. Yijun Wu, Kai Kang, and Chang Han performed the experiment. Yijun Wu, Shaobin Wang, Qi Chen, and Yu Chen contributed significantly to analysis and manuscript preparation. Yijun Wu and Kai Kang performed the data analyses and wrote the manuscript. Chang Han, Shaobin Wang, and Zhikai Liu helped to perform the analysis with constructive discussions.

## ETHICS APPROVAL AND CONSENT TO PARTICIPATE

This study has been approved by the Ethics Committee of Peking Union Medical College Hospital and all patients have signed informed consent.

## CONSENT FOR PUBLICATION

Not applicable.

## Data Availability

The data that supports the findings of this study are available from the corresponding author upon reasonable request.
